# Supplementary figures and images for: Genome-wide characterization and expression of the TLP gene family associated with Colletotrichum gloeosporioides inoculation in Fragaria × ananassa
Source: PeerJ. 2022 Mar 24;10:e12979. doi: 10.7717/peerj.12979 (PMC8958966; doi:10.7717/peerj.12979)

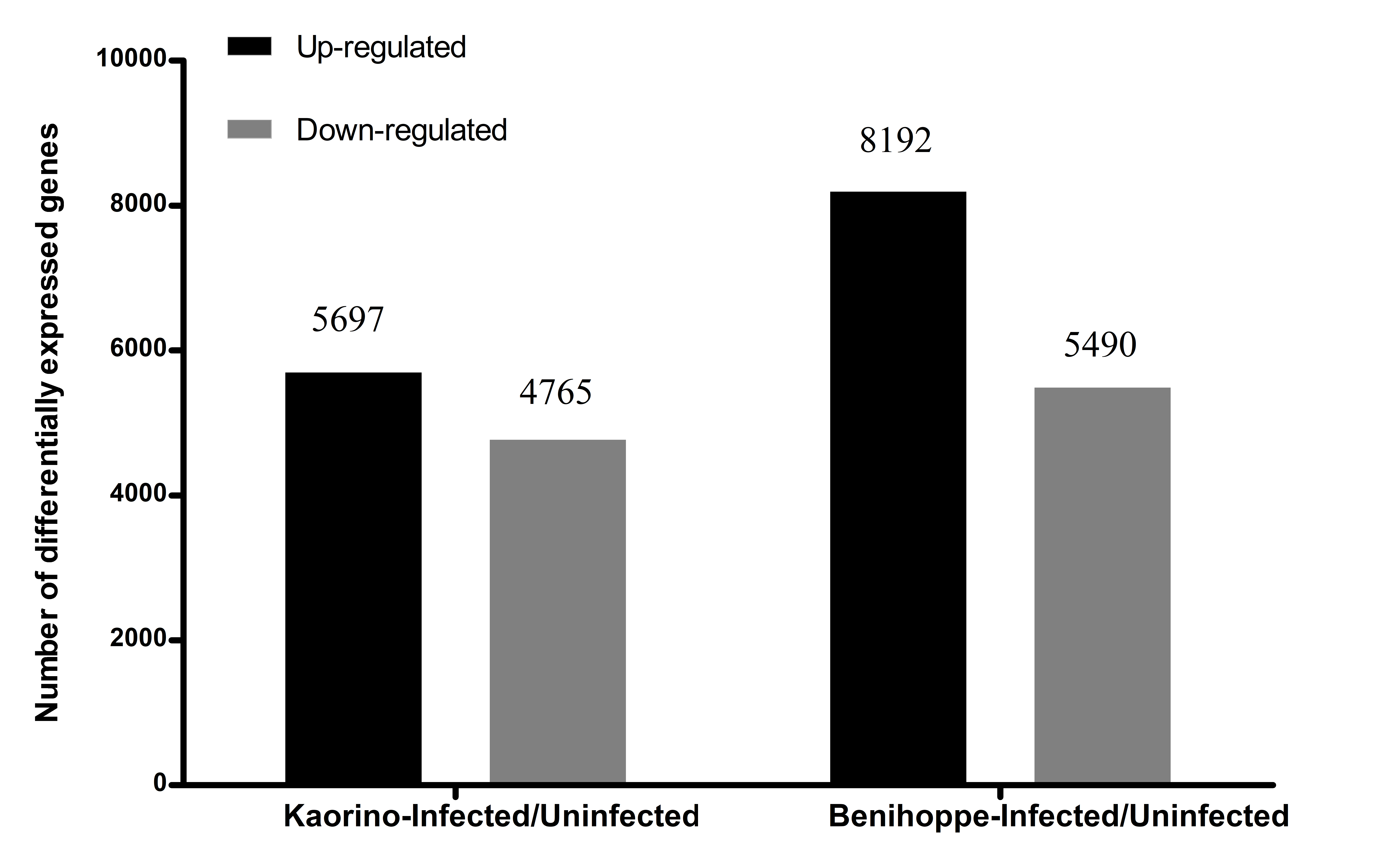

Supplement: Supplemental Information 6 — The x-axis represents the different treatments. The y-axis indicates the number of DEGs. Black and gray sections represent upregulated and downregulated genes, respectively. [file peerj-10-12979-s006.jpg]

### Kaorino-Infected/Uninfected

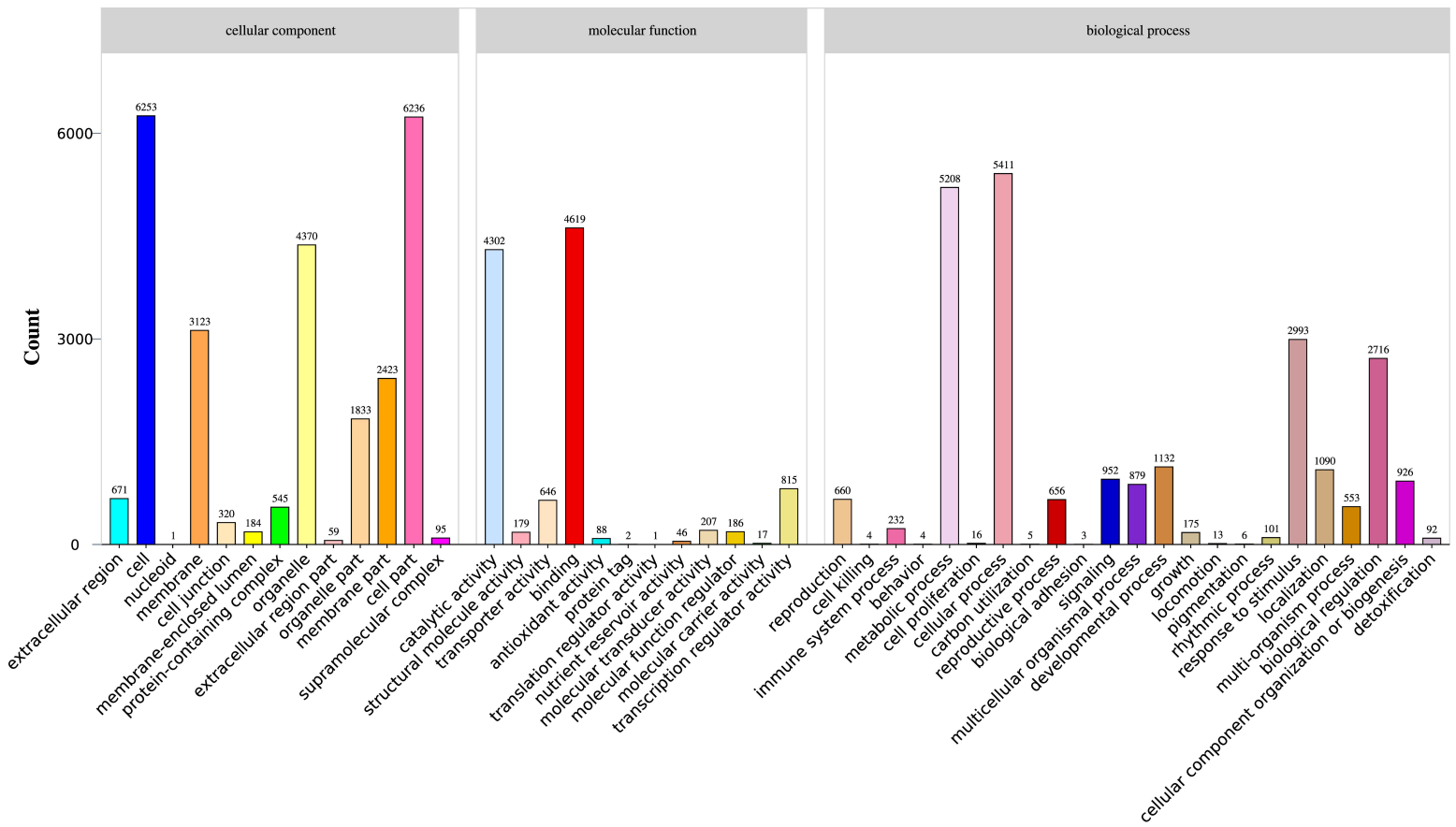

## Benihoppe-Infected/Uninfected

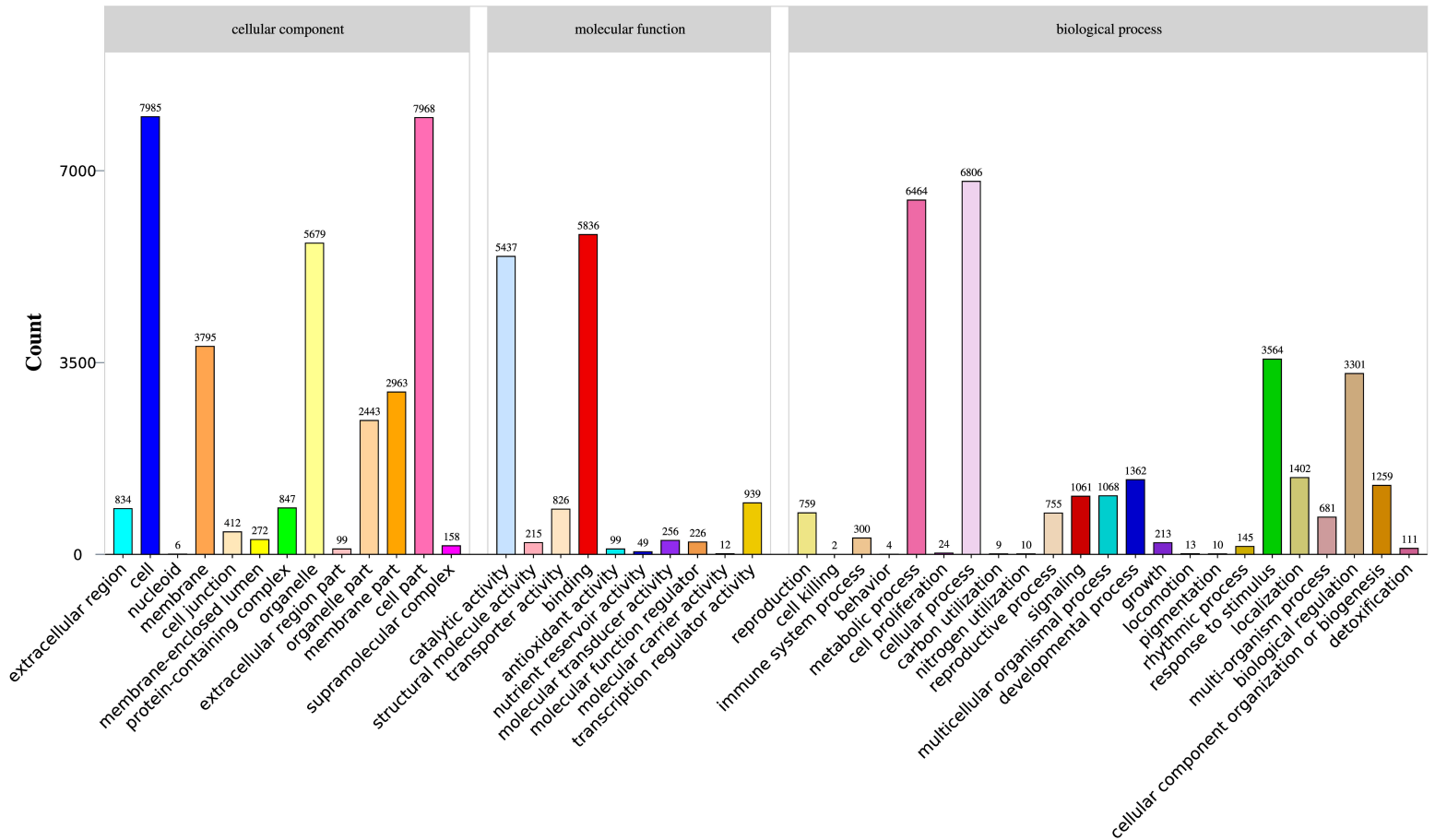

Supplement: Supplemental Information 7 — The x-axis represents enriched GO processes, and different colors represent different GO processes. The y-axis indicates the total number of genes annotated to each GO process. [file peerj-10-12979-s007.pdf]
